# RECIPROCAL RELATIONSHIP BETWEEN SENSE OF PURPOSE AND COGNITIVE CHANGE IN OLDER ADULTHOOD: A COORDINATED ANALYSIS

**DOI:** 10.1093/geroni/igad104.1027

**Published:** 2023-12-21

**Authors:** Gabrielle Pfund, Bryan James, Patrick Hill, Jason Hassenstab, Payton Rule, Emily Willroth

**Affiliations:** Northwestern University, Chicago, Illinois, United States; Rush University Medical Center, Chicago, Illinois, United States; Washington University in St. Louis, St. Louis, Missouri, United States; Washington University in St. Louis, St. Louis, Missouri, United States; Washington University in St. Louis, St. Louis, Missouri, United States; Washington University in St. Louis, St. Louis, Missouri, United States

## Abstract

Sense of purpose (i.e., the extent to which one has personally meaningful goals and directions guiding them through life) is a consistent promoter of healthy aging for older adults. Unfortunately, while individuals benefit from having a high sense of purpose as they age, many people’s sense of purpose declines during older adulthood. Some evidence suggests that these declines may in part be explained by health declines experienced later in life. With sense of purpose being a powerful predictor of cognitive functioning in older adulthood, the current project evaluates whether this relationship may be more reciprocal in nature. Using a coordinated data analytic approach, this work investigates how sense of purpose and cognitive functioning promote each other and change together as older adults age. Longitudinal data comes from the Health and Retirement Study (N=15,497; 4 waves over 12 years), Memory and Aging Project (N=1,700; up to 15 waves over 15 years), Minority Aging Research Study (N=950; up to 12 waves over 12 years), and Wisconsin Longitudinal Study (N=22,334; 3 waves over 18 years). Based on a series of random intercept cross-lagged panel models, initial results indicate that sense of purpose change precedes cognitive functioning change and vice versa, when accounting for between-person and concurrent within-person associations. Moreover, bivariate latent growth models show that longer-term change in sense of purpose co-occurs alongside changes in cognitive functioning. In this talk, we will discuss how promoting sense of purpose can support successful cognitive aging, and the repercussions of cognitive decline for sense of purpose.

